# Green-adapted spectrophotometric determination of fostemsavir based on selective bromophenol blue extraction; reduction of hazardous consumption using computational calculations

**DOI:** 10.1038/s41598-023-36821-x

**Published:** 2023-06-21

**Authors:** Afnan S. Batubara, Bayan E. Ainousah, Mohammed Gamal, Ahmed A. Almrasy, Sherif Ramzy, Mohammed M. Ghoneim, Ahmed H. Abdelazim

**Affiliations:** 1 Department of Pharmaceutical Chemistry, College of Pharmacy, Umm Al-Qura University, Makkah, 21955 Saudi Arabia; 2 Pharmaceutical Analytical Chemistry Department, Faculty of Pharmacy, Beni-Suef University, Beni-Suef, 62514 Egypt; 3 Pharmaceutical Analytical Chemistry Department, Faculty of Pharmacy, Al-Azhar University, Nasr City, Cairo, 11751 Egypt; 4 Department of Pharmacy Practice, College of Pharmacy, AlMaarefa University, Ad Diriyah, 13713 Saudi Arabia

**Keywords:** Analytical chemistry, Green chemistry

## Abstract

A computationally-assisted and green spectrophotometric method has been developed for the determination of fostemsavir, a recently FDA-approved drug used in combination with antiretroviral drugs to treat multidrug-resistant HIV-1 infection. The method was developed using computational studies and solvent selection based on green chemistry principles. The density functional theory method was employed to identify bromophenol blue as the preferred acid dye for efficient extraction of fostemsavir. The solvent selection process involved a careful evaluation of the green ranking of solvents, which led to the use of water as the solvent. The method involved the extraction of fostemsavir with bromophenol blue to form a yellow ion-pair complex, which exhibited maximally sharp peaks at 418 nm, enabling sensitive visible spectrophotometric determination of fostemsavir in bulk and pharmaceutical preparations. The extraction procedures were optimized, and the method was demonstrated to be sensitive over the concentration range of 2–12 μg/mL fostemsavir. Furthermore, the method was evaluated with respect to green chemistry principles using the analytical eco-scale, the green analytical method index, and analytical greenness metric approach, all of which confirmed that the data obtained by the proposed method were environmentally acceptable.

## Introduction

The greening of analytical methods is a promising challenge that has attracted the attention of researchers around the world. The defined principles of green chemistry investigate and evaluate the sustainability of each chemical process in order to reduce the use of hazardous substances, limit the amount of waste, and improve the safety and health conditions for analysts^1,2^. The contribution of computational chemistry to the principles of green chemistry can promote the development of sustainable processes^3^. Computational chemistry can be used to make clear predictions and help guide experimental design. By reducing the number of possible experimental trials, computational chemistry can also serve as an integrative tool to achieve the goals of green chemistry. This can lead to reduced analysis time, lower energy consumption, and lower costs, ultimately contributing to a more sustainable and environmentally friendly approach to chemical research^4–6^.

As chemical reactions generally occur in a solvent, it is crucial to select a safe solvent and reagent. Controlling the emissions of organic and hazardous materials during chemical reactions is highly recommended. Improper disposal of organic laboratory waste can have significant environmental and health impacts, and can also result in costly disposal. One of the most important factors for environmentally friendly analytical chemistry is to replace solvents with environmentally friendly alternatives and minimize the amount of chemicals consumed during chemical reactions^7–10^.

The spectrophotometric determination of drugs based on the verification of their structural properties or functional groups remains an attractive approach in drug analysis^11–13^. Nitrogen-containing drugs with positively charged protonated sites can react with anionic acid dyes in an acidic buffer solution, forming corresponding yellow ion-pair complexes that can be extracted with suitable solvents. Various acid dyes such as bromothymol blue (BTB), bromophenol blue (BPB), and bromocresol green (BCG) can facilitate the extraction of nitrogen-containing drugs and the formation of yellow ion-pair complexes. This reaction serves as the basis for the spectrophotometric determination of many drugs^12–14^.

**Figure 1:**
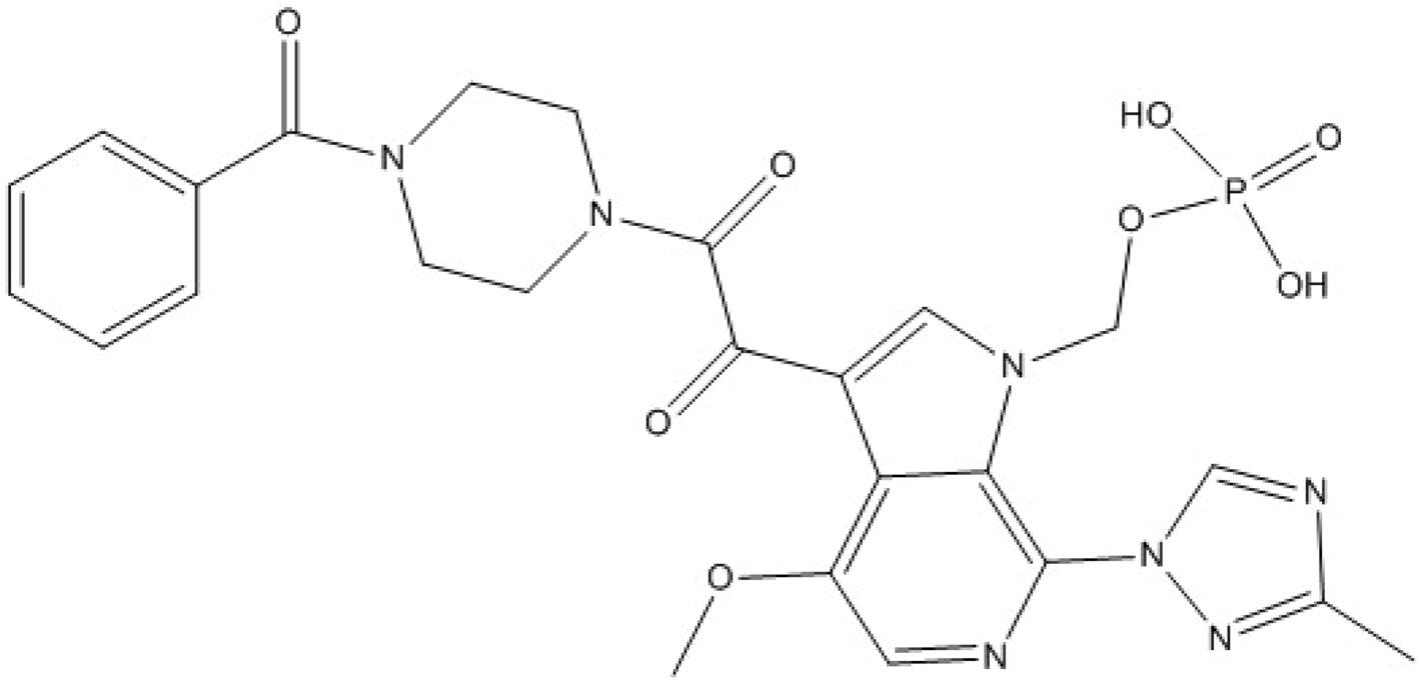
Chemical structure of FV.

Fostemsavir [FV], Fig. 1, is a phosphonooxymethyl prodrug of temsavir, a new HIV-1 attachment inhibitor. It inhibits the activity of gp120, a subunit of the HIV-1 envelope glycoprotein gp160, which prevents the first step in the viral life cycle of HIV-1. FV received FDA approval in July 2020 for use in combination with other antiretroviral drugs for the treatment of multidrug-resistant HIV-1 infection^15,16^.

The current research presents a novel approach by combining computer-assisted studies and green chemistry principles to develop an environmental optimized spectrophotometric method for FV determination. A computer-assisted study was conducted to select the most efficient acid dye for extracting FV, taking into account ecological considerations. Water was chosen as the solvent based on the solubility of FV and its environmental impact. The method involved the use of BPB as the acid dye to extract FV, forming a yellow ion-pair complex that enabled sensitive spectrophotometric determination of FV in bulk and pharmaceutical preparations. The method was also evaluated in terms of green chemistry principles using analytical eco-scale 17, green analytical procedure index^18^, and analytical greenness metric approach [AGREE] evaluation method^19^. The results indicated that the proposed method scored well in terms of green ranking. By integrating calculations and green evaluation metrics, this study presents a promising approach to achieving green goals in analytical chemistry.

## Experimental

### Materials, solvents and reagents

Reference standard pure powder of FV [99.85%] and Rukobia extended-release tablets 600 mg FV were kindly provided by the National Organization for Drug Control and Research, Giza, Egypt.

All chemicals used were kindly provided by El-Nasr Company, Cairo, Egypt. BPB was prepared at concentrations of 0.1% and 1.37 × 10–3 M. Chloroform, carbon tetrachloride, ethyl acetate, hydrochloric acid, methylene chloride, potassium acid phthalate and sodium hydroxide.

### Apparatus

Shimadzu UV–visible 1650 Spectrophotometer, Tokyo, Japan.

### Standard solutions

Standard stock solution of FV [100 μg/mL] was made by dissolving 10 mg of FV the reference standard pure powder in 50 mL water, adjusting at pH 3.7 and the volume was made up to 100 mL with water.

### Procedures

#### Computational testing of extractive acid dye efficiency for FV

Prior to conducting experimental tests, computational calculations were performed to optimize the molecular structure of FV, as well as acid dyes such as BCG, BPB, and BTB, and their corresponding ion-pair complexes, using Gaussian 03 software. The study utilized Density Functional Theory (DFT) and the B3LYP/6-31G (d) basis set^20,21^. The energy of the optimized compounds was measured, and the binding energy of FV with different acidic dyes was determined using the following equation:

$$ \Delta {\text{E }} = {\text{ E}}_{{{\text{A}}{-}{\text{B}}}} - {\text{ E}}_{{\text{A}}} {-}{\text{ n E}}_{{\text{B}}} , $$

where A is the energy of the optimized molecular structure of the FV, B is the energy of the optimized molecular structure of the acidic dyes and ∆E corresponds to the binding energy.

### Experimental procedures

Aliquots of FV at a concentration of 500 μg/mL, corresponding to 50–300 μg, were transferred to a series of 125-mL separation funnels. To the working solutions, 5 mL of phthalate buffer at pH 2.4 and 4 mL of 0.1% BPB solution were added. The aqueous layer was then brought up to a volume of 15 mL with water and extracted with 25 mL of ethyl acetate. The resulting extracts were collected in 25 mL volumetric flasks and made up to the mark with ethyl acetate. The yellow color developed was measured at 418 nm for absorbance values. To prepare the calibration plot, the absorbance values measured were plotted against each drug concentration in µg/mL to calculate the correlative regression equations. All methods were performed in accordance with relevant guidelines and regulations.

### Procedures for determination of the pharmaceutical dosage form

Ten Rukobia tablets were crushed, weighed, and powdered. The weight of the powder equivalent to one tablet was then transferred to a 100-mL volumetric flask and diluted to 50 mL with water. The resulting solution was shaken carefully, filtered, and made up to 100 mL with water to prepare a stock solution containing FV at a concentration of 6 mg/mL. Five concentrations of the pharmaceutical form were then prepared and tested following the general procedure.

## Results and discussion

In this research, an environmentally friendly spectrophotometric method was developed for the determination of FV using computational studies and solvent selection based on green chemistry principles. FV, a newly marketed nitrogenous drug with positively charged protonated sites, was chosen due to its rational structural behavior. The use of theoretical calculations reduced chemical consumption and analysis time. The method was based on the extraction of FV using the anionic acid dye BPB, which formed a yellow ion-pair complex with FV. The absorption spectra of the complex showed maximum peaks at 418 nm, allowing for routine analysis of FV in bulk and pharmaceutical dosage form. The environmental friendliness of the method was also evaluated. This method represents a promising step towards more sustainable analytical practices.

### Solvent selection regarding to green ranking

Substituting hazardous reagents with less hazardous or non-hazardous alternatives is a crucial aspect of workplace risk management. Conducting environmental impact assessments of organic substances is highly recommended due to their direct impact on public health and the environment^22^. To choose a sustainable solvent, we reviewed the available solvents for the drug in question and considered data-rich solvent selection guidelines. The selection was based on three criteria: health, safety, and environmental evaluations for each solvent.

The results showed that the developed method exhibited similar extraction efficiency for FV when using methanol, acetonitrile, and a mixture of methanol and water, as compared to the method using water as the extraction solvent. However, the use of methanol and acetonitrile as the extraction solvent may not be considered as environmentally friendly and associated with health and environmental concerns. Therefore, the use of water as the extraction solvent remains the most environmentally friendly option for the developed method.

### Evaluation of the obtained calculation results for the theoretical testing of different extractive acid dyes

It is essential for researchers to prioritize the principles of green chemistry when conducting chemical processes. While there have been efforts to minimize the adverse effects of chemicals on both health and the environment, there is still more that can be done. The generation of waste from different types of reactions in chemistry laboratories contributes to the depletion of various reagents, some of which may be unsafe and have negative impacts on health and the environment^7,8^. To promote green chemistry, researchers should intensify their search for alternative solvents and reagents that are safer and more environmentally friendly. By carefully selecting these alternatives for each chemical reaction, it is possible to develop efficient and sustainable processes.

Prior to conducting experiments, computer calculations can provide predictive data before the actual experiments are conducted^23–25^. Several theories, including quantum and classical mechanics, semi-empirical structure–property relationships, and DFT, have been described to determine the electronic structure of compounds^6^. DFT derives the actual properties of compounds depending on the evaluation of electron density^20,21^.

To measure the bonding between FV and the acid dyes BTB, BPB and BCG, the calculation DFT was developed to select the best acid dye that allows efficient extraction of FV. Calculations were performed at the B3LYP/6-31G (d) level, which provides a good prediction of binding energy. Medium size 6-31G basis sets were chosen as they are suitable for reaction chemistry calculations.

Geometric optimization of the bonds is an essential step in measuring the corresponding energy values. The binding energy for the complexes formed between FV and the acid dyes was measured, Table 1. The results showed that the binding with BPB could be the most efficient. Based on the obtained data, BPB was theoretically selected and should allow efficient spectrophotometric determination of FV in the visible range.

**Table 1:** Pre-experimental computer-aided study of the binding energy between FV and various acid dyes.

Name of complex	Interaction energy	
	∆E (Hartree)^a^	∆E (KJ/mol)
FV-BPB	**− 0.00801**	**− 21.03**
FV-BCG	**− **0.00452	**− **11.86
FV-BTB	**− **0.00203	**− **5.33

Significant values are in bold.

^a^1Hartree = 2625.5 kJ/mol.

### Spectral characteristics

**Figure 2:**
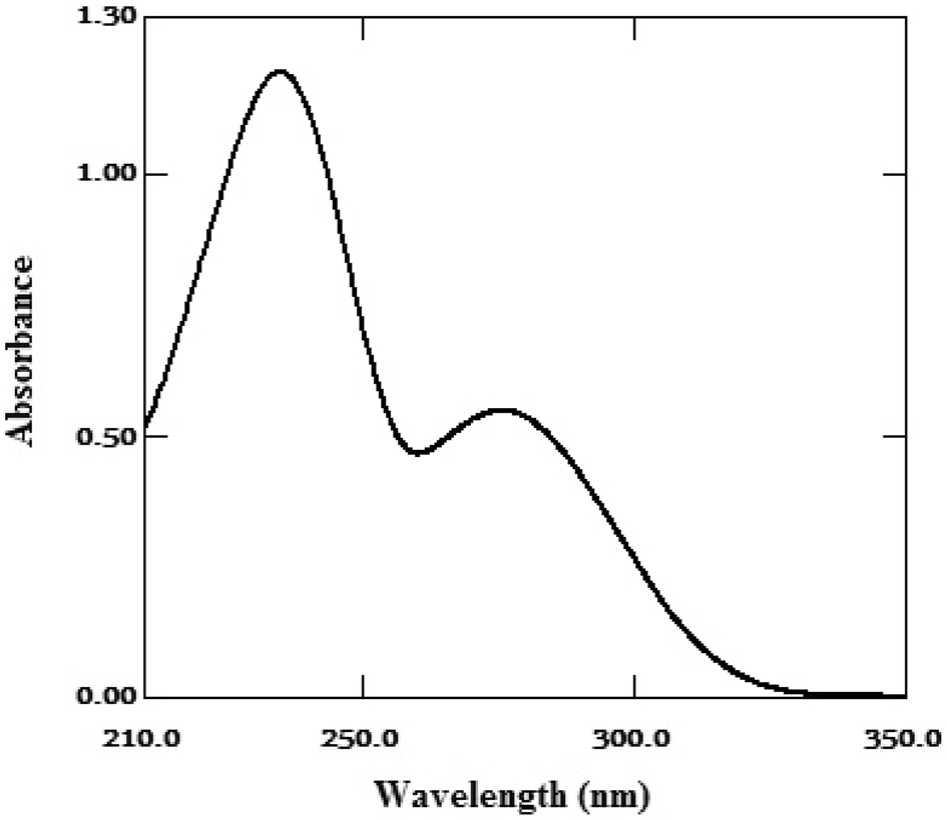
The absorption spectra of FV (20 µg/mL).

**Figure 3:**
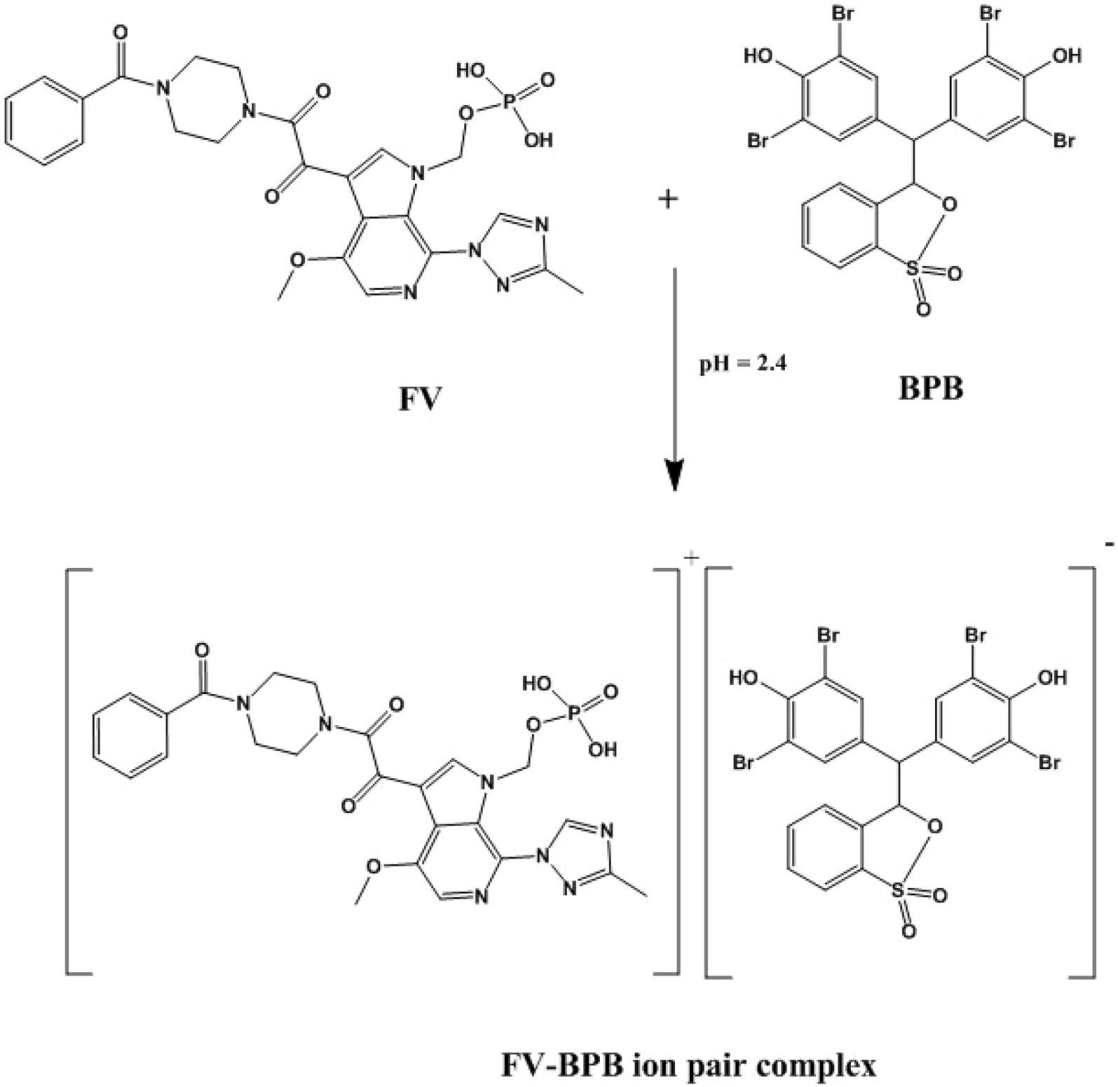
The proposed schematic diagram for the reaction between FV and BPB.

**Figure 4:**
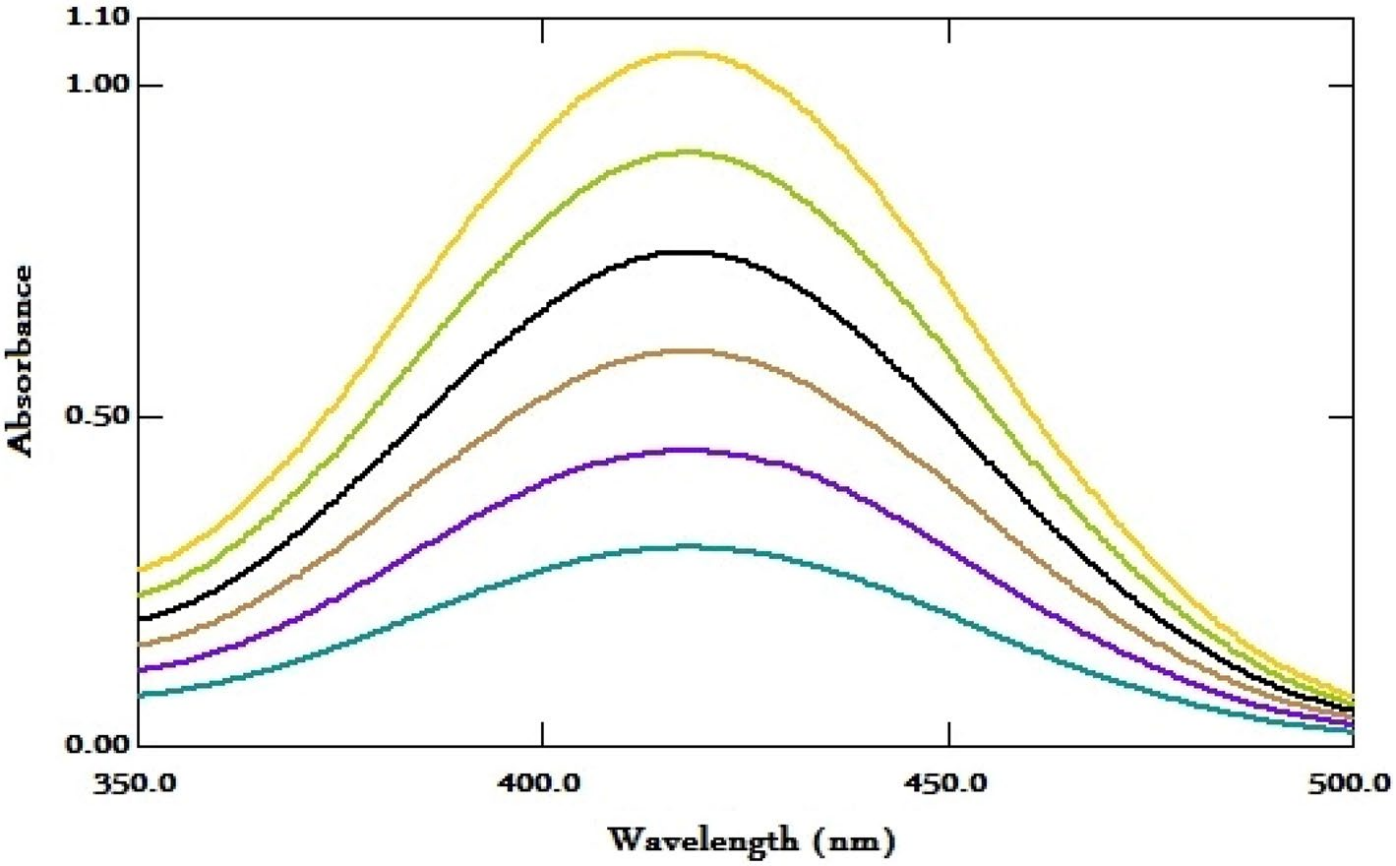
Absorbance spectra of the obtained yellow ion-pair complex FV-BPB (2–12 µg/mL).

The nitrogen-containing drug, FV, contains positively charged protonated sites, which makes it prone to forming a yellow-colored ion-pair complex when it interacts with a negatively charged dye in an acidic environment. This interaction causes a shift in the electronic structure of the dye, resulting in a change in the absorption spectrum of the complex. The complex can be extracted using a suitable solvent and measured spectrally. FV was found to have a maximum UV absorption peak at 278 nm, Fig. 2 and was extracted using BPB to form the corresponding yellow ion-pair complex, as depicted in the schematic reaction pathway in Fig. 3. The absorption spectra of the obtained ion-pair complex showed maximum peaks at 418 nm, Fig. 4, which enabled the determination and routine analysis of FV in both bulk and pharmaceutical dosage forms.

### Optimization of the experimental conditions

**Figure 5:**
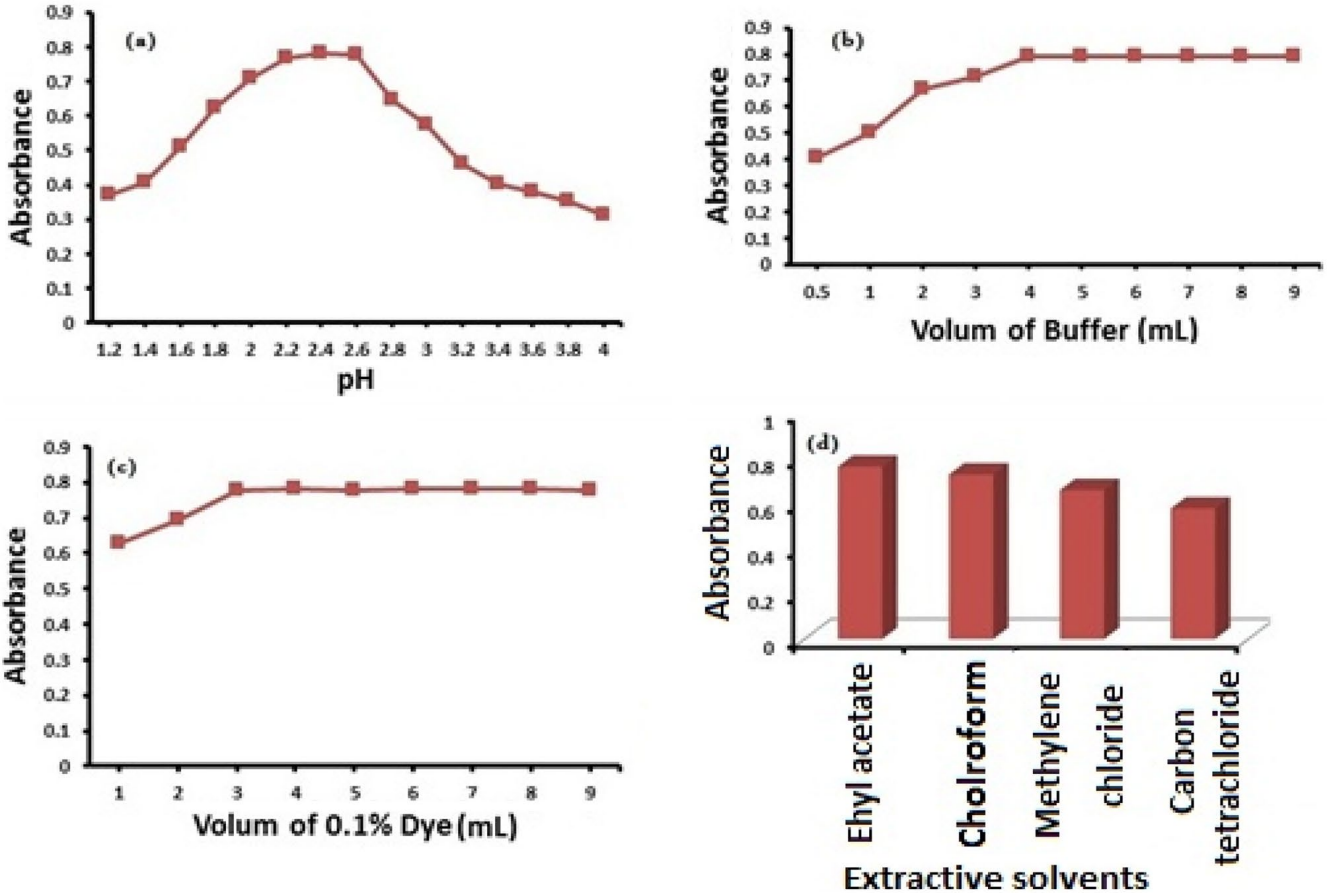
Optimization for different factors affecting the reaction of FV with BPB.

Optimization of reaction conditions was carefully considered to ensure efficient extraction of the mentioned drug. The effect of pH on the FV-BPB complex was tested by extracting the colored complex product in the presence of different buffer solutions. The maximum color intensity and the highest absorbance value were observed when phthalate buffer with pH 2.4 was used. Moreover, 5.0 mL of the phthalate buffer gave maximum absorbance values and reproducible results. The results of the buffer test are shown in Fig. 5a,b.

The effect of BPB volume was verified by evaluating the absorbance of solutions with a fixed concentration of FV and different amounts of BPB. The maximum color intensity of the complex was observed when 4 mL of 0.1% BPB was used. The result of the BPB volume test is shown in Fig. 5c.

To ensure efficient extraction of the colored ion-pair complex from the aqueous phase, the effect of different organic solvents was tested. 25 mL ethyl acetate proved to be a suitable solvent for the extraction of the FV-BPB ion-pair complex, as shown in Fig. 5d.

### Stoichiometric relationship

**Figure 6:**
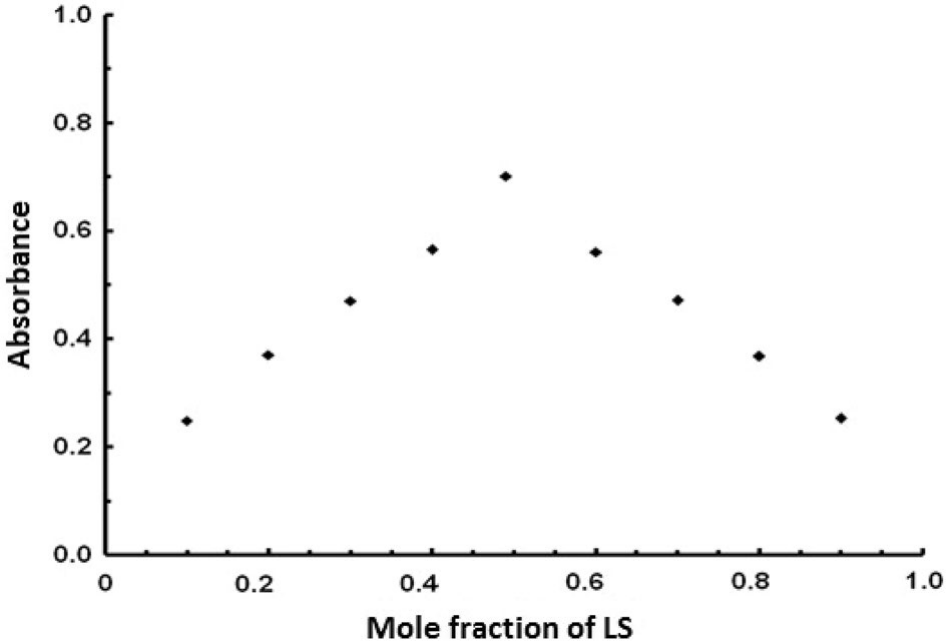
Stoichiometry of the reaction of [1.37 × 10^–3^ M] FV with [1.37 × 10^–3^ M] BPB by Job’s method of continuous variation.

The stoichiometric ratio for the reaction of FV and BPB was determined using Job's continuous variation method^26^. The graph of absorbance values at 418 nm versus molar fraction FV was plotted as shown in Fig. 6. The results showed that 1:1 [FV: BPB] ion pair complexes were formed by the electrostatic attraction between positive protonated FV and negative anionic BPB.

### Validation of the method

The described method was validated following the guidelines of ICH. Linearity was evaluated by analyzing serially prepared solutions of FV in the concentration range of 2–12 μg/mL using the proposed method. The regression data are presented in Table 2.

**Table 2:** Parameters of the proposed spectrophotometric determination of FV.

Parameters		FV
Wavelength (nm)		418
Linearity range (μg/mL)		2–12
Regression values		
Slope		0.0410
Intercept		0.0012
Coefficient of determination (r^2^)		0.9998
LOD (μg/mL)		0.10
LOQ (μg/mL)		0.30
Accuracy (%R)^a^		99.08
Precision (%RSD)^b^	Repeatability	0.523
	Intermediate precision	0.626
Robustness, (% R ± %RSD)	Detection wavelength (± 2 nm)	99.87 ± 0.681
	pH (± 0.2)	100.49 ± 0.982
Pharmaceutical preparation (%Recovery ± %RSD)^c^		99.0 ± 0.83

^a^Values of 9 determinations (3 concentrations repeated 3 times).

^b^Values of 9 determinations (3 concentrations repeated 3 times).

^c^Values for five determinations of the pharmaceutical preparation.

The limit of detection (LOD) and limit of quantification (LOQ) of the method were estimated based on the residual standard deviation of a regression line (σ) and the slope (S) using the following equations:

$$ {\text{LOD }} = { 3}.{3 }\sigma /{\text{S,}} $$

$$ {\text{LOQ }} = { 1}0 \, \sigma /{\text{S}}{.} $$

The LOD and LOQ of the method were determined to be 0.10 μg/mL and 0.30 μg/mL, respectively.

Accuracy was determined by applying the described method to triplicate determinations of FV at concentrations of 3, 6, and 9 µg/mL. The mean percent recovery (%R) was calculated and found to be acceptable, as presented in Table 2. Precision, expressed as percent relative standard deviation (%RSD), was determined by analyzing FV at concentrations of 3, 6, and 9 µg/mL. For repeatability, the analysis was performed within one day, and for intermediate precision, it was performed on three consecutive days. The lower values of %RSD indicated higher precision of the described method, as shown in Table 2.

The specificity of the method was determined by applying the standard addition technique to check the effect of the tablet matrix. Known quantities of FV in pure form were added to already analyzed pharmaceutical preparation, and %R of the pure added concentrations was calculated. The obtained results, as presented in Table 3, indicated that the proposed method could selectively analyze the drug without any interference from excipients.

**Table 3:** Specificity study of the proposed method using standard addition technique.

	Pharmaceutical taken (µg/mL)	Pharmaceutical found (µg/mL)	Pure added (μg/mL)	Pure found (µg/mL)	%R
FV	5	4.94	3	3.01	100.33
			5	5.08	101.60
			7	7.11	101.57
Mean ± %RSD					101.16 ± 0.687

Method robustness indicates its efficiency to remain unaffected by small but deliberate variations in the method parameters. It was assessed by calculating %RSD through repeating the procedures under slight changes in the described conditions, such as detection wavelength (± 2 nm) and pH (± 0.2). The obtained %RSD values revealed method robustness, as shown in Table 2.

The stability of the method was evaluated by analyzing FV solutions stored at room temperature and under refrigeration for 24 and 48 h. The measured concentrations were compared with the initial concentrations to determine the stability of the method. The results showed that the method was stable for at least 48 h under both storage conditions, with less than 2% deviation from the initial concentration. Therefore, the method was deemed to be stable for the duration of the analysis.

### Determination of FV in pharmaceutical dosage form

The described method was successfully applied for the determination of FV in bulk and pharmaceutical dosage form. The results, Table 2, showed satisfactory recovery data, and the test results for the pharmaceutical form also agreed well with the described method.

#### Green evaluation of the described method

Research attention has been paid toward green chemistry approaches^27–31^. To evaluate and rank the greenness of the described method, the analytical eco-scale score was used^17^. Solvent consumption was measured mainly as a function of the amount of solvent consumed. The analytical eco-scale score provides data that reflect the environmental impact of the analytical method. The calculated score was 81, indicating that the analytical method is environmentally friendly and has minimal negative impact on the environment and human health. Furthermore, the green analytical method index^18^ presents a precise tool to consider the different variables of the analytical procedure, including sample preparation, sample handling (collection, preservation, transport, and storage), and the chemicals and instrumentation used. Each variable was colored from green to yellow to red, indicating low, medium, or high negative environmental impact, respectively. The described method had five green zones and one red zone. The results demonstrated the environmentally friendly nature of the developed method, as evidenced by the green assessment results. Finally, the AGREE tool^19^ was used, which presents the environmental friendliness profile of the analytical methods as a numerical value. The obtained value was 0.82, confirming the green characteristics of the developed method. In summary, the results of the green metrics presented a detailed environmental friendliness profile and confirmed compliance with environmentally friendly practices in most cases. The green assessment results are presented in Table 4.

**Table 4:** Green evaluation of the described method.

Parameter	Data obtained
National environmental method index	
Green analytical procedure index	
Novel analytical greenness metric	

### Comparative assessment of the proposed method and previously reported HPLC method

The proposed spectrophotometric method was compared to a previously published HPLC method^32^ in terms of sensitivity, detection limits, and quantification. The current method allowed for the determination of FV over a concentration range of 2–12 μg/mL. The lower detection limit of 0.10 μg/mL and quantitation limit of 0.30 μg/mL confirmed that the introduced method was more sensitive than the previously published method. Moreover, the described spectrophotometric method is more environmentally friendly as it uses water as a solvent instead of acetonitrile, which is toxic and hazardous. The method also requires less expensive equipment and has a shorter analysis time (Table 5)

**Table 5:** Comparative assessment of the proposed method and previously reported HPLC method.

	Proposed spectrophotometric method	Published HPLC method^32^
Principle	Acidic dye extraction and formation of ion-pair complex	Liquid chromatography
Solvent	Water (environmentally friendly)	Acetonitrile (toxic, hazardous)
Equipment	Visible spectrophotometer	HPLC
Detection	Absorbance at 418 nm	UV detection at 278 nm
Sensitivity (μg/mL)	2–12	15–90
LOD (μg/mL)	0.10	0.18
LOQ (μg/mL)	0.30	0.54

## Conclusion

In this study, we presented an environmentally-friendly spectrophotometric method for the determination of fostemsavir, which was developed using computational studies and solvent selection based on green chemistry principles. The computational study revealed that bromophenol blue was an efficient extractant for fostemsavir. The method involved the extraction of fostemsavir with bromophenol blue, an acidic dye, resulting in the formation of a yellow ion-pair complex with sharp absorption peaks at 418 nm. The method was evaluated in terms of its adherence to green chemistry principles, and the results demonstrated its superior environmental friendliness compared to previously published methods.

## Data Availability

The datasets used during the current study are available from the corresponding author on reasonable request.
